# Non-Linear Center-of-Pressure Features Associated with Fall History in Older Adults: An Exploratory Analysis

**DOI:** 10.3390/s26082298

**Published:** 2026-04-08

**Authors:** Dai Wakabayashi, Yohei Okada

**Affiliations:** 1 Graduate School of Health Sciences, Kio University, Nara 635-0832, Japan; w.dai.0216@gmail.com; 2 Nishiyamato Rehabilitation Hospital, Nara 639-0218, Japan; 3 Neurorehabilitation Research Center, Kio University, Nara 635-0832, Japan

**Keywords:** center of pressure, postural sway, fall, older adults, loss of complexity, non-linear dynamics, multiscale entropy, recurrence quantification analysis, SHAP

## Abstract

Postural sway derived from center-of-pressure (CoP) trajectories is widely used to assess balance and fall risk in older adults, but conventional linear metrics mainly quantify sway magnitude and may overlook temporal organization. Guided by the loss-of-complexity hypothesis, we re-examined associations between fall history and linear and non-linear CoP metrics in an open-access dataset. Quiet-standing trials under eyes-open and eyes-closed conditions were analyzed in adults ≥60 years (fallers *n* = 19; non-fallers *n* = 57). To reduce confounding, propensity score matching was performed using age, sex, body mass index, activities of daily living level, illness status, number of medications, disability status, and orthosis/prosthesis use. Linear and non-linear indices, including recurrence quantification analysis, detrended fluctuation analysis, fractal dimension, multiscale entropy, stabilogram diffusion analysis, and sway density measures, were examined. After matching, no CoP metric differed significantly between groups. However, SHAP-based exploratory analysis suggested that non-linear features related to temporal structure and multiscale organization contributed more prominently to model output than conventional magnitude-based metrics. Given the limited sample size, these findings should be interpreted as exploratory and hypothesis-generating.

## 1. Introduction

Falls in older adults are a major public health issue because they frequently lead to injury, reduced mobility, and loss of independence. Since impaired postural control is a key contributor to falls, quantitative assessment of postural sway using force plates [1,2] and wearable inertial sensors [3,4] has long been used for fall-risk evaluation. Force-plate measurements during quiet standing provide a practical means of quantifying postural sway using center-of-pressure (CoP) time series, and numerous studies have examined associations between CoP-based metrics and fall risk. However, a persistent challenge is that conventional linear sway metrics—such as sway range, mean velocity, total path length, and sway area—primarily capture the magnitude of CoP fluctuations and do not consistently differentiate fallers from non-fallers across studies and experimental conditions [5,6,7,8]. This inconsistency suggests that fall-related postural impairment may not be fully explained by sway “amount” alone. Mechanistically, CoP reflects whole-body dynamics arising from interactions among multiple joints and sensory–motor processes; therefore, similar sway magnitudes can emerge from different control strategies. As a result, analyses focusing solely on magnitude or variability may overlook how postural control is organized over time.

To address this limitation, non-linear approaches have increasingly been introduced to characterize the temporal structure of CoP fluctuations [9,10]. These methods quantify features such as complexity, regularity, and long-range organization, which may reflect adaptive control rather than mere instability [11,12,13,14,15]. According to the loss-of-complexity hypothesis, healthy physiological systems exhibit complex fluctuations across multiple time scales, whereas aging and pathology often reduce this complexity, leading to more regular and less adaptive dynamics. This concept was originally proposed by Lipsitz and Goldberger and has since been widely applied to studies of physiological variability and aging. Importantly, “complexity” in this context does not simply indicate larger sway or greater variance; instead, it refers to structured variability that is neither overly rigid nor completely random. Accordingly, both excessively stereotyped patterns and overly random fluctuations may indicate reduced adaptability, underscoring the need to evaluate not only sway magnitude but also the integrity of its temporal organization [16,17,18,19,20].

In this study, we re-examine postural sway in older adults from the perspective of the loss-of-complexity hypothesis, considering both its magnitude and temporal structure. Using an open-access CoP dataset, we compare older adults classified as fallers and non-fallers. In addition to conventional linear sway metrics, we compute a range of non-linear indices, including recurrence quantification analysis (RQA), detrended fluctuation analysis (DFA), fractal dimension (FD), multiscale entropy (MSE), stabilogram diffusion analysis (SDA), and the sway density curve (SDC), under the same dataset and task conditions. This perspective suggests the hypothesis that non-linear measures may capture aspects of postural control that are not reflected in conventional magnitude-based sway metrics. However, previous studies have often focused on individual nonlinear metrics or specific analytical approaches [13]. Few studies have systematically evaluated multiple nonlinear descriptors alongside conventional magnitude-based sway metrics within the same dataset and task conditions. In particular, it remains unclear whether nonlinear indices capture complementary aspects of postural control beyond conventional sway measures during relatively low-demand quiet-standing tasks. By integrating conventional magnitude-based measures and temporal-structure-based non-linear measures within a single analytical framework, this study aims to examine whether non-linear indices provide complementary information beyond traditional sway metrics. Rather than proposing a clinically deployable classifier, this secondary analysis adopts an exploratory analytical framework to examine patterns related to fall history and to provide a unified perspective on postural dynamics in older adults.

## 2. Materials and Methods

### 2.1. Participants

This study is a secondary analysis of an open-access postural control database developed by Santos and Duarte (2016) [21]. The dataset has been widely used in postural control research, supporting its methodological reliability [22,23,24,25]. The original dataset included 163 community-dwelling adults (116 females, 47 males) aged 18–85 years recruited from a Brazilian university and the surrounding community.

The original study excluded individuals unable to safely perform the static standing task or those with severe impairments significantly affecting balance function (e.g., major vestibular, visual, musculoskeletal, or cognitive impairments). Detailed sociodemographic characteristics, self-reported medical history, medication status, and presence of disabilities are documented in the metadata file (BDSinfo) accompanying the database.

Participants aged ≥60 years were selected to focus on fall risk in older adults. Eligibility criteria included complete stabilometry records from 60-s static standing trials on a firm surface under both eyes-open and eyes-closed conditions, as well as available fall history data within the past 12 months (Falls12m). Based on Falls12m, participants were classified into two groups: fallers (Falls12m ≥ 1) and non-fallers (Falls12m = 0). Falls12m is a retrospective self-reported count of non-intentional falls over the prior 12 months. Information about fall circumstances (e.g., balance-related vs. accidental/environmental causes) is not available in this dataset.

The intergroup comparison in this study was conducted between these two subgroups to examine the relationship between postural control indicators and fall risk among older adults. Written informed consent was obtained from all participants during the original data collection, and the study protocol was approved by the Ethics Committee of the Federal University of ABC (UFABC; Universidade Federal do ABC), Brazil (approval number 842529/2014). Since this study used only anonymized public data and did not involve new data collection, no additional ethical approval was required in accordance with the guidelines of the affiliated institution.

### 2.2. Equipment

In the original data-collection study, static standing was assessed using a 40 × 60 cm force plate (OPT400600-1000; AMTI, Watertown, MA, USA) connected to an Optima Signal Conditioner (AMTI). The force plate was factory-calibrated and had an average CoP accuracy of 0.02 cm; measurement precision, estimated from the standard deviation of the CoP signal during a 30 s recording with a 30 kg static load, was 0.005 cm. Force (Fx, Fy, Fz) and moment (Mx, My, Mz) signals were acquired at 100 Hz using NetForce software (Version 3.5.3; AMTI). The dataset providers computed the center of pressure (CoP) in the anteroposterior (CoP_AP) and mediolateral (CoP_ML) directions from these signals. Analyses were conducted using the provided CoP time series in both the anteroposterior and mediolateral directions for all trials from the included participants.

### 2.3. Procedure

The stabilometry protocol followed the procedures described by Santos and Duarte (2016) [21]. Participants performed a 60-s static standing task under four conditions varying visual input and support surface: (1) firm surface with eyes open, (2) firm surface with eyes closed, (3) foam surface with eyes open, and (4) foam surface with eyes closed. Each condition was repeated three times in a randomized order. According to the original protocol, if a participant could not complete a 60-s trial, the measurement was stopped and the trial was reattempted up to two times; if completion was still not achieved, the corresponding trial was recorded as missing. In the present older-adult subset (Age ≥ 60, *n* = 76), foam-condition trials were missing for a small number of participants: three participants lacked foam trials under both visual conditions, and one lacked the eyes-closed foam condition only. Because the public dataset does not specify the reasons for missing trials, it is not possible to determine whether these were due to task difficulty or other factors.

Participants stood barefoot on the force plate with their arms at their sides and were instructed to remain as still as possible. Foot position was standardized with a foot angle of 20° and a heel-to-heel distance of 10 cm, marked on the platform. For the eyes-open condition, participants fixated on a black circular target (5 cm in diameter) affixed to a wall 3 m ahead. For the eyes-closed condition, participants first fixated on the same target, then assumed a comfortable standing posture before closing their eyes, at which point measurement began. All measurements were conducted in a controlled laboratory environment (approximately 4.5 × 2.8 m) with white walls and adequate illumination, as described in the original dataset, ensuring consistent testing conditions.

The present study restricted analysis to the firm-surface eyes-open and eyes-closed conditions in older adults. All available 60-s trials were included for each participant and condition. Firm-surface standing is considered more suitable for detecting intrinsic postural-control differences, whereas foam conditions primarily assess compensatory responses to external instability; therefore, these conditions were selected to evaluate fall-risk–related postural control in older adults.

### 2.4. Data Analysis

All analyses were performed using MATLAB (v2022) (MathWorks, Natick, MA, USA) with custom scripts developed for this study. Anteroposterior (AP) and mediolateral (ML) center-of-pressure (CoP) coordinates were obtained from the public dataset, which had been recorded using a force platform manufactured by Advanced Mechanical Technology, Inc. (AMTI, Watertown, MA, USA). The CoP time series were low-pass filtered using a second-order, zero-phase Butterworth filter with a cutoff frequency of 20 Hz, applied to 60-s recordings (6000 samples) sampled at 100 Hz. Instantaneous CoP velocity was calculated by differentiating the filtered CoP signals and multiplying by the sampling frequency.

Linear sway metrics were first calculated to characterize the magnitude and variability of postural sway. These included mean planar CoP velocity (Velo), the area of the 95% confidence ellipse of the CoP trajectory (Area), direction-specific mean velocities (Velo AP, Velo ML), and standard deviations of CoP velocity (Velo SD AP, Velo SD ML). In addition, velocity-derived mean frequency indicators (MFREQ AP, MFREQ ML) were calculated as the ratio of mean CoP velocity to the mean distance from the spatial center [26].

Non-linear metrics were then computed to characterize the temporal organization and spatial complexity of CoP sway beyond amplitude-based measures alone. Summary indices derived from the sway density curve (MP3, MT3, MD3) were calculated as compact descriptors of postural stabilization behavior [27]. The fractal dimension (FD) of the planar CoP trajectory was estimated using a length-dependent algorithm equivalent to the Katz method [28]. Long-range temporal correlation properties were evaluated using detrended fluctuation analysis (DFA), yielding scaling exponents for the AP and ML directions (α_AP, α_ML) [29]. Stabilogram diffusion analysis (SDA) was performed on the planar CoP trajectory to calculate short- and long-term diffusion coefficients (SDA Ds, SDA Dl), as well as the critical time and distance at which diffusion behavior transitions between regimes (SDA CriT, SDA CriD) [30]. To assess the complexity of CoP dynamics across multiple time scales, multiscale entropy (MSE) analysis was conducted using sample entropy with embedding dimension m = 2 and tolerance r = 0.2 times the standard deviation. Entropy values at scales 1, 10, and 40 were extracted for both the AP and ML directions (MSE1, MSE10, MSE40) [31].

Finally, recurrence quantification analysis (RQA) was applied to state-space reconstructed CoP time series in both directions. Time delay and embedding dimension were determined using auto mutual information and false nearest neighbors methods, respectively [32,33]. The recurrence threshold was set to yield a recurrence point density of approximately 5%, following prior recommendations for postural sway analysis [34]. Because the present study used a relatively small sample, we restricted RQA to two commonly reported and comparatively interpretable indices—determinism (DET) and laminarity (LAM)—to limit feature proliferation and reduce overfitting risk while retaining measures relevant to regularity and intermittency of postural sway [34]. For MSE and RQA calculations, functions based on analysis code shared in the Non-linear-Analysis-Core (NONANLibrary) were used. All computed CoP-derived variables were treated as candidate predictors in the subsequent exploratory multivariate analyses, together with conventional linear sway measures, to examine their relative contributions to fall-history classification. To help readers visualize the analyzed signal and reconstruction process and improve transparency of the analytical pipeline, a representative example of the raw CoP trajectory, corresponding ML and AP time series, reconstructed state-space trajectory, and recurrence plot is provided in Supplementary Figure S1.

### 2.5. Statistical Analysis

All statistical analyses were performed using R statistical software (R version 4.4.1; R Core Team, 2024, R Foundation for Statistical Computing, Vienna, Austria). CoP indices were averaged across the available 60-s trials within each condition to improve measurement reliability and reduce within-participant variability.

Propensity scores were estimated using a logistic regression model with fall status (Falls12m ≥ 1 vs. 0) as the outcome. Based on a priori clinical relevance and data availability, age, sex, body mass index (BMI), activities of daily living (ADL) level, illness status, number of medications, disability status, and orthosis/prosthesis use were included as covariates in the propensity score model [35]. One-to-one nearest-neighbor matching without replacement was then performed using a caliper width of 0.2 times the standard deviation of the logit-transformed propensity scores [36]. Covariate balance before and after matching was assessed using standardized mean differences (SMDs) and visually examined using Love plots. The estimand was the average treatment effect on the treated (ATT), with fallers defined as the treatment group. Because some residual imbalance remained after matching, particularly for age, the propensity score–matched analysis was interpreted as a sensitivity analysis rather than definitive evidence of complete covariate balance. After matching, group differences in CoP indices were evaluated using Welch’s *t*-test. Given the number of comparisons and the exploratory aim of the univariate analyses, these comparisons were interpreted descriptively. In addition, covariate-adjusted and propensity score–stratified analyses were performed to examine the robustness of the findings. Statistical significance was set at a two-tailed *p* < 0.05.

To examine the multivariate contribution of CoP metrics to fall-history classification, logistic regression models with standardized predictors were constructed. Logistic regression was selected because, in a relatively small sample, it provides a parsimonious and interpretable multivariate framework with a lower risk of overfitting than more flexible classifiers. Because propensity score matching substantially reduced the available sample size, the exploratory multivariate analyses were conducted using the original dataset to retain statistical power. Feature contributions were assessed using approximate Shapley values (SHAP) computed with the fastshap algorithm [37]. In the present study, SHAP was used as a model-based contribution analysis to visualize the relative direction and magnitude of feature contributions at the model-output level, rather than as direct evidence of mechanistic interpretation. To minimize optimistic bias and prevent information leakage, we employed a repeated stratified 5-fold cross-validation procedure repeated 30 times, yielding 150 training/test splits per condition. Within each training set, SHAP values were computed using the training data only, and features were ranked accordingly. The outer test folds were reserved exclusively for out-of-fold evaluation.

Given the retrospective and potentially heterogeneous nature of the fall outcome and the limited number of recurrent fallers, the primary aim of the multivariate analyses was not to develop a deployable classifier, but to explore whether non-linear CoP metrics capture patterns related to fall history beyond conventional magnitude-based sway measures. Accordingly, model performance metrics are reported as exploratory estimates rather than definitive predictive accuracy.

## 3. Results

### 3.1. Propensity Score Matching and Intergroup Differences in CoP Metrics

**Figure 1:**
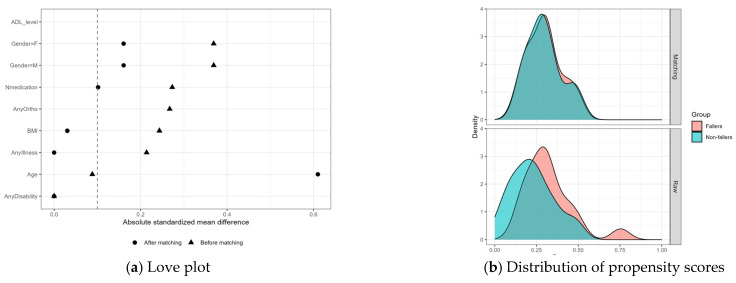
Propensity score matching summary: (**a**) Love plot showing absolute standardized mean differences before and after matching for the covariates included in the propensity score model. The dashed vertical line indicates the commonly used threshold of 0.1 for acceptable balance. (**b**) Distribution of propensity scores in fallers and non-fallers before matching (raw sample) and after matching. One-to-one nearest-neighbor matching yielded 18 matched pairs. Although propensity score overlap improved after matching, residual imbalance remained for some covariates, particularly age.

To examine the potential influence of measured confounding, a propensity score–matched sensitivity analysis was performed in older adults. The baseline characteristics of the original dataset are summarized in Table 1 (19 fallers and 57 non-fallers). Propensity score matching yielded 18 matched pairs (matched sample: 18 fallers and 18 non-fallers). Among participants aged ≥60 years, the number of recurrent fallers (Falls12m ≥ 2) was very small (*n* = 3). Propensity score overlap improved after matching; however, some residual imbalance remained, particularly for age, indicating that matching did not fully eliminate potential confounding (Figure 1a,b).

**Figure 2:**
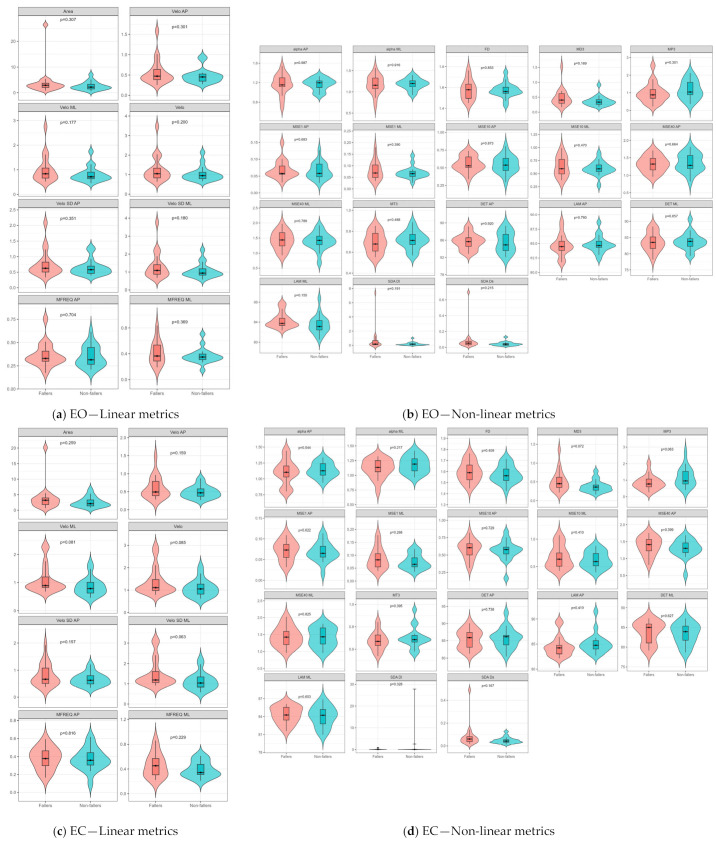
Violin plots of linear and non-linear CoP sway metrics after propensity score matching: Distributions of CoP measures are shown for the eyes-open condition (EO; linear metrics, (**a**); non-linear metrics, (**b**) and the eyes-closed condition (EC; linear metrics, (**c**); non-linear metrics, (**d**)). Pink and blue violins represent fallers and non-fallers, respectively. Inner boxplots indicate the interquartile range, and the central line denotes the median. Welch’s *t*-test *p*-values for comparisons between fallers and non-fallers are shown above each metric. No metric reached statistical significance after matching (all *p* > 0.05).

After propensity score matching, Welch’s *t*-tests revealed no statistically significant differences between fallers and non-fallers in any linear or non-linear CoP metric under the eyes-open condition (all *p* > 0.05) (Figure 2a,b). Visual inspection of the violin plots showed substantial overlap between groups across most measures. Similar findings were observed under the eyes-closed condition, with no significant group differences detected for any CoP metric (Figure 2c,d). Overall, these findings suggest that, after accounting for measured background factors, no single CoP metric showed a robust univariate association with fall history in this matched sample.

### 3.2. SHAP-Based Feature Contribution Analysis

**Figure 3:**
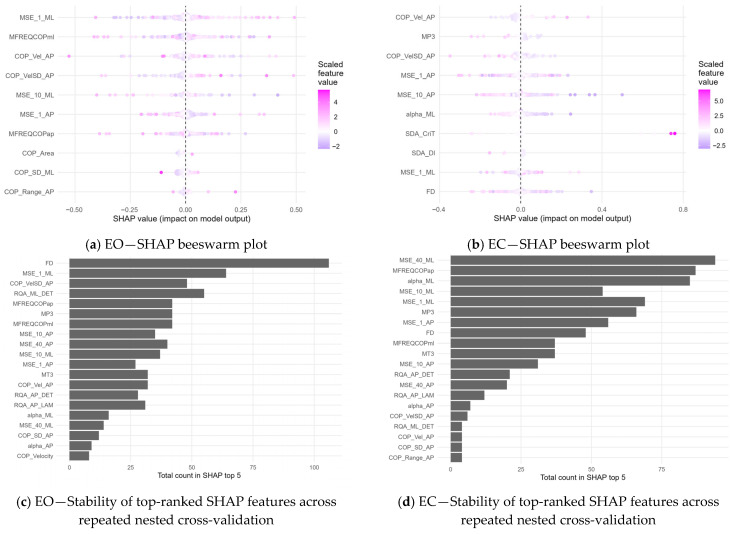
SHAP-based feature contribution and stability: In panels (**a**,**b**), each dot represents the SHAP value of one subject, and the horizontal axis indicates the direction and magnitude of each feature’s contribution to the model output. Features are ordered according to their mean absolute SHAP values. Dot color indicates the scaled feature value, with lower values shown in cooler tones and higher values shown in warmer tones. In panels (**c**,**d**), bars indicate the proportion of repeated runs in which each feature appeared among the top 5 features ranked by mean absolute SHAP value.

SHAP analysis indicated that non-linear CoP features were relatively prominent contributors to fall-risk classification under both the eyes-open and eyes-closed conditions (Figure 3).

Under the eyes-open condition (Figure 3a), features related to entropy, frequency characteristics, and CoP velocity contributed prominently to model output. In particular, MSE_1_ML showed the largest mean absolute SHAP value, followed by MFREQCOPml, COP_Vel_AP, and COP_VelSD_AP, suggesting that short-timescale mediolateral irregularity and anteroposterior sway dynamics were important contributors to classification. Additional non-linear features, including entropy- and complexity-related indices, also showed meaningful contributions.

Under the eyes-closed condition (Figure 3b), a similar overall pattern was observed, with non-linear and dynamic CoP features remaining frequently represented among influential features. In this condition, COP_Vel_AP, MP3, COP_VelSD_AP, MSE_1_AP, and MSE_10_AP were among the most influential features, indicating that both sway dynamics and temporal structure showed notable contributions to model predictions.

The stability analysis further showed that the top-ranked SHAP features were relatively stable across repeated nested cross-validation, particularly in the eyes-open condition (Figure 3c). In the eyes-closed condition (Figure 3d), several leading features also remained stable, although lower-ranked features showed greater variability across repetitions.

## 4. Discussion

This study suggests that fall-related information in older adults may be embedded in the temporal organization of postural sway rather than in sway magnitude alone. By systematically examining multiple nonlinear descriptors alongside conventional sway metrics within the same dataset and task conditions, the present study provides an integrated perspective on how the temporal organization of postural sway may relate to fall history, particularly in low-demand quiet-standing tasks where conventional magnitude-based measures often show limited group separation. Using a well-characterized open-access force-platform dataset and an analytical framework integrating conventional linear and non-linear CoP metrics, univariate analyses adjusted for confounding factors revealed no significant differences between fallers and non-fallers during quiet standing on a firm surface. In contrast, interpretable multivariate analyses indicated that non-linear indicators reflecting temporal structure and multi-timescale characteristics contributed prominently within exploratory models beyond conventional magnitude-based measures. Collectively, these findings suggest that fall-related information may not manifest as mean differences in single indicators but instead as composite patterns reflecting the “quality” of postural variability. Importantly, these findings should not be interpreted as evidence that nonlinear CoP metrics are inherently superior to conventional sway measures, but rather that they may capture complementary aspects of postural dynamics that are not fully reflected by magnitude-based descriptors alone.

### 4.1. Background for the Absence of Univariate Differences and the Role of Non-Linear Complexity

The absence of significant univariate differences after propensity score matching (PSM) underscores the difficulty of explaining fall risk using a single CoP indicator. CoP represents the integrated output of multi-joint and multi-sensory postural control (visual, vestibular, and somatosensory inputs). Accordingly, distinct control strategies may yield similar sway amplitudes. When postural sway is summarized solely by global descriptors (e.g., mean velocity or sway area), between-group differences in control organization may be attenuated or obscured. In addition, this work is a secondary analysis of a public dataset. To prioritize methodological consistency and reproducibility across conditions, we restricted analyses to the standard quiet-standing task on a firm surface. Whether non-linear indicators exhibit stronger group separation under more challenging task constraints remains an important question for future investigation.

The present results suggest that fall-history–related differences may be reflected in the temporal organization and multi-scale structure of postural sway than in its magnitude alone. Because postural control emerges from multi-sensory integration and multi-joint coordination, similar sway magnitudes may arise from different underlying control strategies. From this perspective, MSE may reflect complexity across multiple time scales, RQA indices may capture regularity and persistence in the underlying control dynamics, FD may reflect the geometric complexity of the trajectory, DFA may index long-range temporal correlations, and SDA-derived measures may reflect transitions between different control regimes. These indices may therefore complement conventional quantitative measures such as velocity and area by capturing qualitative alterations in postural control dynamics that are less accessible through amplitude-based descriptors alone. Importantly, these interpretations are grounded in the established dynamical properties of these indices and should not be interpreted as direct mechanistic inference from SHAP feature rankings. This interpretation is broadly in line with the loss-of-complexity hypothesis, which suggests that aging and pathology are associated with simplification of multiscale physiological variability, resulting in reduced adaptability. Because the present study is based on a small-sample exploratory analysis, these findings should not be interpreted as evidence that non-linear metrics are dominant, but rather as hypothesis-generating observations supporting the relevance of temporal structure in postural control.

SHAP analyses further suggested condition-specific patterns. Under eyes-open (EO) conditions, non-linear features such as short-scale multiscale entropy (e.g., MSE1 in the AP direction), fractal dimension (FD), and RQA measures related to local stability and regularity were ranked highly. Under eyes-closed (EC) conditions, longer-timescale MSE components (e.g., MSE10–MSE40 in the ML direction), FD, and detrended fluctuation analysis (DFA) scaling exponents (α in the ML direction) were relatively prominent. These features describe temporal organization—regularity, local stability, long-range correlations, and multi-scale structure—rather than oscillation magnitude. The relatively frequent retention of MSE, DFA, and RQA features in the multivariate models is therefore consistent with the possibility that fall-history–related information may be associated with alterations in the structure of postural variability (i.e., the “quality” of sway), rather than with amplitude-based measures alone. However, SHAP quantifies feature contributions within a multivariate modeling framework and does not establish causal relationships. Accordingly, these interpretations should be regarded as mechanistic hypotheses requiring further targeted validation.

### 4.2. Clinical Implications

These findings suggest that non-linear CoP analyses can complement conventional sway metrics by providing additional insights into the organization of postural control. Importantly, fall-related information may reside not only in the magnitude of sway but also in the structure of postural variability, indicating that assessment strategies focusing solely on amplitude-based measures may overlook clinically relevant features.

At the same time, the present results suggest that fall-related information may not be fully captured by any individual CoP metric, but may become more apparent when conventional and non-linear features are considered together. This perspective is consistent with the view that alterations in postural control may reflect multiple interacting aspects of postural sway dynamics rather than a single dominant marker [13].

The prominence of long-timescale features under eyes-closed conditions further highlights the potential role of sensory integration and adaptive control processes when visual information is unavailable. Although immediate clinical implementation remains premature, these results support the conceptual value of incorporating complexity-oriented metrics into postural-control assessment frameworks.

For clinical translation, standardization of measurement protocols and reproducible feature computation will be essential. Although the present findings are not sufficient to support immediate clinical implementation, they suggest the potential value of integrating conventional measures (e.g., velocity and area) with a parsimonious set of robust non-linear indicators, thereby enhancing sensitivity to subtle alterations in postural control while preserving clinical interpretability.

### 4.3. Limitations and Future Perspectives

Several limitations should be noted. First, the sample size was limited, particularly after propensity score matching, and the dataset was imbalanced in terms of fall status. These constraints reduce statistical power and increase the risk of overfitting and unstable multivariate estimates, limiting the robustness and generalizability of the findings. Non-linear measures often exhibit higher variability, which may further reduce sensitivity to detect group differences in small samples; therefore, the absence of statistically significant differences in non-linear metrics may, at least in part, reflect limited statistical power rather than a true lack of association. Therefore, the multivariate results should be interpreted as exploratory and hypothesis-generating rather than clinically predictive.

Second, fall status was derived from a retrospective self-reported count of non-intentional falls over the prior 12 months (Falls12m) and may therefore be subject to recall bias. Moreover, the number of recurrent fallers (Falls12m ≥ 2) was extremely small in the present older-adult subset, which precluded meaningful subgroup analyses based on fall recurrence.

Third, information on the specific circumstances or causes of falls was not available in the public dataset. Therefore, it was not possible to distinguish falls related to balance impairment from incidental, accidental, or environmental events. This lack of information on the causes of falls limits the interpretability of associations between postural sway measures and fall history and the generalizability of the findings beyond the present dataset.

Fourth, although propensity score matching was performed using available background variables, residual confounding cannot be excluded. While matching improved comparability between groups, unmeasured or incompletely controlled factors—such as cognitive status, physical performance, comorbidities, and habitual physical activity—may still have influenced both fall history and postural control. For this reason, the present matched analysis should be interpreted as exploratory rather than as definitive evidence of causal or fully adjusted group differences.

Finally, the present analyses were restricted to quiet standing on a firm surface to ensure methodological consistency when comparing conventional and non-linear CoP features. Because foam-surface standing imposes greater sensory and motor demands, the exclusion of these conditions limits generalizability to more challenging balance tasks. In addition, a small number of older participants had missing foam-condition trials, which may indicate that individuals with more severe balance impairment were underrepresented. Accordingly, the present findings, derived from an exploratory analysis of a specific subset, may not generalize to older adults with different clinical profiles, including those with recurrent falls, greater frailty, more severe balance impairment, or task-specific instability under more challenging sensory conditions.

Future studies should therefore evaluate these non-linear indicators using prospective designs with larger and more balanced cohorts, clearer operational definitions of falls, and a broader range of postural tasks, including more challenging standing conditions, to clarify their robustness, clinical relevance, and mechanistic significance. Based on the present exploratory patterns, future prospective studies may particularly benefit from testing parsimonious combinations of conventional sway measures and temporal-structure-based non-linear metrics, rather than evaluating each metric in isolation. Candidate combinations include velocity-based descriptors (e.g., mean CoP velocity, velocity variability, and MFREQ) together with multiscale entropy measures (especially short- and intermediate-scale MSE components), sway density indices such as MP3, and selected regularity-related RQA features, which may together capture complementary aspects of postural control.

## 5. Conclusions

This study suggests that fall-related information in older adults may be reflected not solely in the magnitude of postural sway but also in the temporal organization of postural variability. While conventional sway metrics showed limited separation between fallers and non-fallers under low-demand quiet-standing conditions, SHAP-based interpretation of multivariate models suggested that non-linear features capturing multi-timescale dynamics were relatively prominent contributors in exploratory analyses beyond traditional measures.

These findings support a complexity-oriented perspective on postural assessment and suggest that integrating non-linear indicators with conventional sway metrics may provide additional insight into fall-related postural dynamics. However, given the limited sample size and class imbalance, these results should be interpreted as exploratory and hypothesis-generating in nature. Future studies using larger and more balanced cohorts, as well as prospective designs and more challenging task conditions, will be necessary to clarify the clinical and mechanistic relevance of non-linear CoP dynamics.

## Figures and Tables

**Table 1:** Characteristics of the participants.

Group	N	Age (Years)	BMI (kg/m^2^)	Body Height (cm)
Non-fallers	57	71 (6.3)	25.7 (2.9)	157.9 (8.7)
Fallers	19	71 (7.1)	25.0 (2.8)	155.4 (6.1)

Values are presented as mean (SD). Falls were defined as non-intentional events in the past 12 months (self-reported). Participants were classified as Fallers (≥1 fall) or Non-fallers (0 falls).

## Data Availability

Publicly available datasets were analyzed in this study. The data can be accessed via PhysioNet (https://doi.org/10.13026/C2WW2W) and Figshare (https://doi.org/10.6084/m9.figshare.3394432).

## Supplementary Materials

The following supporting information can be downloaded at: https://www.mdpi.com/article/10.3390/s26082298/s1, Figure S1: Representative example of the original CoP data and the recurrence analysis procedure. (a) Two-dimensional CoP trajectory in the mediolateral (ML) and anteroposterior (AP) directions during a representative standing trial. (b) Corresponding ML and AP time-series signals. (c) Reconstructed state-space trajectory obtained from the CoP time series using time-delay embedding. (d) Recurrence plot derived from the reconstructed state-space trajectory. This figure is provided to help readers visualize the analyzed signal and the reconstruction process underlying the RQA measures used in the study.
