# Supplementary material for: Non-Linear Center-of-Pressure Features Associated with Fall History in Older Adults: An Exploratory Analysis
Source: Sensors (Basel). 2026 Apr 8;26(8):2298. doi: 10.3390/s26082298 (PMC13119576; doi:10.3390/s26082298)
Supplement: Supplementary file 1 [file sensors-26-02298-s001.zip › sensors-4184047-supplementary.pdf]

Supplementary Figure S1

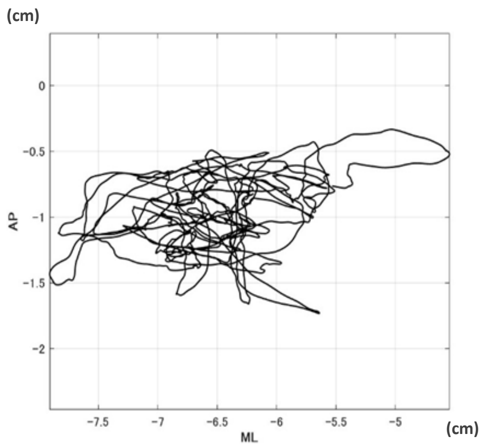

(**a**) CoP trajectory in the ML–AP plane

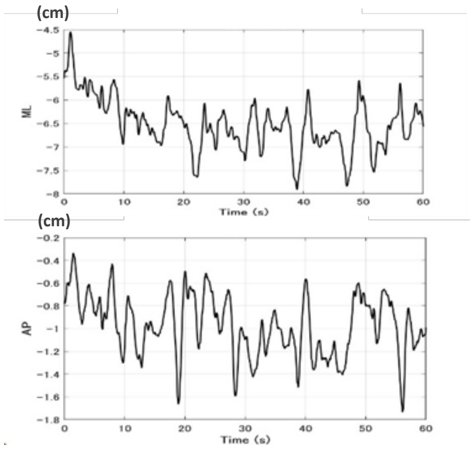

(**b**) ML and AP time series

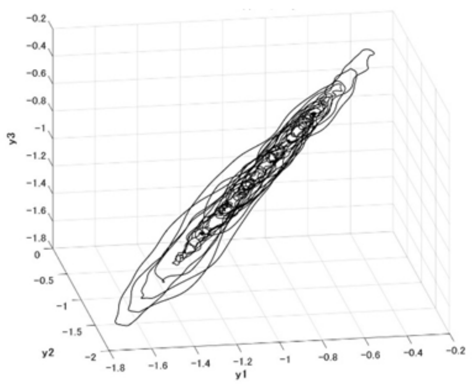

(c) Reconstructed state-space trajectory

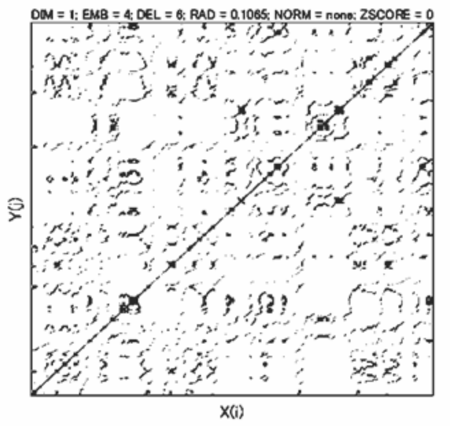

(d) Recurrence plot

Supplementary Figure S1. Representative example of the original CoP data and the recurrence analysis procedure. (a) Two-dimensional CoP trajectory in the mediolateral (ML) and anteroposterior (AP) directions during a representative standing trial. (b) Corresponding ML and AP time-series signals. (c) Reconstructed state-space trajectory obtained from the CoP time series using time-delay embedding. (d) Recurrence plot derived from the reconstructed state-space trajectory. This figure is provided to help readers visualize the analyzed signal and the reconstruction process underlying the RQA measures used in the study.
